# Analysis of Predictive Factors for Cognitive Function Improvement After tDCS Treatment in Patients With Post-stroke Cognitive Impairment

**DOI:** 10.62641/aep.v54i2.2161

**Published:** 2026-04-15

**Authors:** Ting Li, Jixin Lin, Liya Zhang, Xin Wang, Jie Zhang, Jing Xu, Qing Ye

**Affiliations:** ^1^Department of Rehabilitation Medicine, Center for Rehabilitation Medicine, Rehabilitation & Sports Medicine Research Institute of Zhejiang Province, Zhejiang Provincial People’s Hospital (Affiliated People’s Hospital), Hangzhou Medical College, 310024 Hangzhou, Zhejiang, China; ^2^Department of Geriatric Psychiatry, The Third People’s Hospital of Fuyang, 310009 Hangzhou, Zhejiang, China

**Keywords:** post-stroke cognitive impairment, transcranial direct current stimulation, cognitive function, predictive factors, Mini-Mental State Examination, Modified Barthel Index

## Abstract

**Objective:**

This study aims to identify predictive factors for significant cognitive improvement following transcranial direct current stimulation (tDCS) in patients with post-stroke cognitive impairment (PSCI), thereby providing evidence for individualized clinical intervention.

**Methods:**

A total of 123 patients with PSCI who received tDCS treatment were retrospectively enrolled. Based on changes in Mini-Mental State Examination (MMSE) scores, patients were classified into a cognitive improvement group (n = 61) and a non-improvement group (n = 62). Baseline clinical characteristics were collected, and activities of daily living were using the Modified Barthel Index (MBI). Univariate analyses were performed to compare differences between the two groups, and variables with statistical significance in univariate analysis were further entered into a multivariate logistic regression model to identify independent predictors of significant cognitive improvement following tDCS treatment.

**Results:**

The proportion of patients with a university education or above was significantly in the cognitive improvement group higher than in the non-improvement group (*p* < 0.001); whereas the proportion of patients with a history of stroke in the non-improvement group was significantly higher (*p* < 0.05). Patients in the cognitive improvement group had a significantly shorter disease duration compared to those in the non-improvement group (*p* < 0.05); meanwhile, a higher proportion of patients with Fazekas grade 0–1 was observed in the improvement group (*p* < 0.05). Results of the multivariate Logistic regression analysis indicated that educational level and disease duration were independent predictive factors for significant cognitive improvement after tDCS treatment (*p* < 0.05).

**Conclusion:**

PSCI patients with higher educational level and shorter disease duration have a better cognitive improvement effect following tDCS treatment.

## Introduction

Stroke is one of the leading causes of mortality and disability worldwide. Post-stroke cognitive impairment (PSCI) is a common complication following stroke, with an estimated incidence ranging from 20% to 80% [1]. Patients with PSCI present with deficits across multiple cognitive domains, including memory, attention, executive function, and language, which significantly impair activities of daily living and quality of life, thereby imposing a substantial burden on both families and society [2, 3]. Currently, treatment options for PSCI remain limited, primarily including pharmacological therapies and cognitive rehabilitation training [4, 5, 6]; however, their clinical efficacy remains unsatisfactory [7]. Therefore, identifying safe and effective treatment modalities as well as, elucidating predictors of treatment response is of paramount importance for improving patient outcomes.

Transcranial direct current stimulation (tDCS) is a non-invasive neuromodulation technique that alters cortical excitability and neuroplasticity by delivering a low-intensity current through scalp electrodes, thereby influencing cognitive function [8]. In recent years, tDCS has attracted increasing attention as a potential intervention for PSCI. Accumulating evidence suggests that tDCS, particularly when targeting cognition-related brain regions such as the left dorsolateral prefrontal cortex (DLPFC), can enhance cognitive performance in PSCI patients by promoting the release of key neurotransmitters, including dopamine and acetylcholine [9, 10]. Nevertheless, not all PSCI patients experience significant cognitive improvement following tDCS. A central challenge is lack of well-established predictors of treatment response, hindering the ability to “precisely identify the most suitable candidates” for tDCS in clinical practice. Consequently, identifying predictive factors that predict therapeutic efficacy and stratifying patients most likely to benefit from tDCS have emerged as critical research priorities aimed at enabling precision medicine in PSCI management.

This study retrospectively analysed clinical data from PSCI patients who received tDCS treatment to explore baseline characteristics associated with significant cognitive improvement. The findings aim to provide evidence-based guidance for individualized clinical decision-making.

## Methods

### Participants

Patients with PSCI admitted between January 2024 and June 2025 were retrospectively enrolled.

Inclusion criteria: (1) Diagnosis of acute stroke confirmed by Magnetic Resonance Imaging (MRI), meeting established diagnostic criteria for stroke [11]; (2) Clinically stable after acute stroke management, with stable vital signs, clear consciousness, and no aphasia; (3) Age ≥18 years; (4) Mini-Mental State Examination (MMSE) score between 10 and 26 within 6 months after stroke onset [12]. (5) Two weeks before treatment and during the treatment period, the medication was stable, with no new, adjusted or deleted drugs related to cognitive function.

Exclusion criteria: (1) History of intellectual disability, dementia, or other psychiatric disorders; (2) Contraindications to tDCS, including skull defects in the stimulated area, intracranial metallic implants, or a history of epilepsy; (3) Patients who declined to participate in the study.

Based on changes in MMSE scores following tDCS treatment, patients were divided into two groups: The cognitive improvement group (n = 61), defined as an increase in MMSE score >3 points from baseline; The non-improvement group (n = 62), defined as an increase in MMSE score ≤3 points or a decrease from baseline [13, 14, 15]. The study was approved by the Zhejiang Provincial People’s Hospital ethics review board (Approval No.: 2025-285) and conducted in strict compliance with the principles outlined in the Declaration of Helsinki. All participants provided written informed consent before inclusion.

### Baseline Data Collection

Baseline data were extracted from the electronic medical record system, including demographic characteristics (age, sex, educational level), past medical history (history of hypertension [systolic blood pressure ≥140 mmHg and/or diastolic blood pressure ≥90 mmHg [16], or previously diagnosed and treated with medication], history of diabetes mellitus [fasting blood glucose ≥7.0 mmol/L or 2-hour postprandial blood glucose ≥11.1 mmol/L [17], or previously diagnosed and treated with medication], smoking history [cumulative smoking ≥100 cigarettes, and currently smoking or quit smoking <1 year], history of alcoholism [n (%)] [weekly alcohol intake ≥140 g of pure alcohol for at least ≥1 year], history of stroke (yes, no), family history [first-degree relatives with stroke or cognitive impairment]), disease-related indicators (disease duration [time from stroke onset to the start of tDCS treatment, days], brain injury location [classified as left brain, right brain, brainstem, and multiple locations based on cranial MRI], stroke type [ischemic/hemorrhagic], Fazekas grading [18] of white matter lesions [double-blindly evaluated by 2 neuroradiologists: Grade 0 = no lesions; Grade 1 = punctate lesions; Grade 2 = patchy confluent lesions; Grade 3 = extensive confluent lesions]), and activities of daily living (assessed using the Modified Barthel Index [MBI] [19], which comprises 10 items including feeding, personal hygiene, dressing, toileting, ambulation, and climbing stairs, the total score of 0–100 points; with higher score indicates better activities of daily living).

### tDCS Treatment Procedures

All patients received treatment using the same tDCS device (Model A620P, Volcan Medical Technology Co., Ltd., Nanjing, Jiangsu, China). Treatment parameters followed international guidelines [20, 21]: The F3 region was selected as the left DLPFC according to the 10–20 EEG system. The anode electrode was placed over the DLPFC, while the cathode over the contralateral (right) orbital region; stimulation was set at an intensity of 2 mA for 20 minutes per session, administered five times per week for 4 consecutive weeks (total of 20 sessions). All sessions were conducted under the supervision of a qualified rehabilitation physician.

### Cognitive Function Assessment

Cognitive function was evaluated by experienced rehabilitation therapists using the MMSE before and after the tDCS treatment. The MMSE comprises five domains: orientation (to time and place), memory (immediate and delayed recall), attention and calculation, language (naming, repetition, reading comprehension), and visuospatial ability (drawing). The total score ranges from 0 to 30, with higher scores indicating better cognitive function [22].

### Statistical Analyses

Statistical analyses were performed using SPSS version 26.0 (IBM Corp., Armonk, NY, USA). The continuous variables were assessed using the Shapiro-Wilk method. Normally distribution data are presented as mean ± standard deviation ($\bar{x}$ ± s), and skewed distribution variables are expressed as median (25% and 75% quartiles). Comparisons between groups were conducted using the independent-samples *t*-test. Categorical variables are expressed as frequencies (percentages) [n (%)], with between-group comparisons performed using the chi-square (χ^2^) test or Fisher’s exact test when expected cell counts were less than 5. Variables with a *p* value < 0.1 in the univariate analyses were included in a multivariate logistic regression model using the stepwise method to identify independent predictors of significant cognitive improvement. A two-sided *p* value < 0.05 was considered statistically significant.

## Results

### Demographic Characteristics

The proportion of patients with a university education or above in the cognitive improvement group was higher than that in the non-improvement group (*p* < 0.001); the proportion of patients with a history of stroke who had no significant cognitive improvement after tDCS treatment (*p* < 0.05). No statistically significant differences in age, gender distribution, or other medical histories between the two groups (*p* > 0.05), as shown in Table 1.

**Table 1.** **Comparison of demographic characteristics between the two groups**.

Variables		Improvement group (n = 61)	Non-Improvement group (n = 62)	T/χ²	*p* value
Age (years, $\bar{x}$ ± s)		59.98 ± 14.73	63.11 ± 14.81	1.175	0.243
Gender [n (%)]				1.542	0.214
	Male	42 (68.85)	36 (58.06)		
	Female	19 (31.15)	26 (41.94)		
Education level [n (%)]				16.374	<0.001
	Illiterate	6 (9.83)	7 (11.29)		
	Primary school	13 (21.31)	23 (37.10)		
	Middle school	22 (36.07)	29 (46.77)		
	University degree or above	20 (32.79)	3 (4.84)		
History of hypertension [n (%)]		38 (62.29)	42 (67.74)	0.401	0.526
History of diabetes mellitus [n (%)]		14 (22.95)	16 (25.81)	0.136	0.712
Smoking history [n (%)]		21 (34.43)	17 (27.42)	0.707	0.400
History of alcoholism [n (%)]		20 (32.79)	16 (25.81)	0.724	0.395
History of stroke [n (%)]		5 (8.20)	11 (17.74)	8.726	0.033
Family history [n (%)]		8 (13.11)	9 (14.52)	1.060	0.589

### Disease-related Indicators and Activities of Daily Living

The disease duration was significantly shorter in the cognitive improvement group than that in the non-improvement group (*p* < 0.05); the proportion of patients with Fazekas grade 0–1 in the cognitive improvement group was higher than that in the non-improvement group (*p* < 0.05); No statistically significant differences in the distribution of brain injury location, stroke type, or pre-treatment MBI score between the two groups (all *p* > 0.05). Detailed results are presented in Table 2.

**Table 2.** **Comparison of disease-related indicators and MBI scores between the two groups**.

Variables		Improvement group (n = 61)	Non-Improvement group (n = 62)	T/χ²	*p* value
Disease duration (Days, $\bar{x}$ ± s)		25 (13.5–45)	35 (20–65)	2.528	0.013
Brain injury location [n (%)]				2.304	0.743
	Left brain	24 (39.34)	20 (32.26)		
	Right brain	31 (50.82)	34 (54.84)		
	Brainstem	4 (6.56)	3 (4.86)		
	Multiple locations	2 (3.28)	4 (6.45)		
Stroke type [n (%)]				0.076	0.782
	Ischemic	33 (54.10)	32 (51.61)		
	Hemorrhagic	28 (45.90)	30 (48.39)		
Fazekas garde [n (%)]				9.262	0.026
	0–1 garde	54 (88.52)	46 (74.19)		
	2–3 garde	7 (11.48)	16 (25.81)		
MBI score ($\bar{x}$ ± s)		30.90 ± 3.27	37.42 ± 2.00	1.482	0.141

MBI, Modified Barthel Index.

### Multivariate Logistic Regression Analysis of Risk Factors for Cognitive Function Improvement

Variables with *p* < 0.05 in the univariate analysis were included in the multivariate Logistic regression model. In addition, to correct the influence of age on cognitive level, it was also included in the multivariate analysis. Among them, history of stroke (no history of stroke was set as the dummy variable), educational level (dummy variables were set for “Illiterate”) and Fazekas grading of white matter lesions (dummy variable was set for “grade 0–1”) were categorical variables, while age and disease duration was continuous variables. The results showed that educational level and disease duration were independent predictive factors for significant improvement in cognitive function of PSCI patients after tDCS treatment (*p* < 0.05). Detailed results are presented in Table 3.

**Table 3.** **Multivariate Logistic regression analysis**.

	B	SE	Wald	*p* value	OR	95% CI
Age	0.002	0.017	0.011	0.916	1.002	0.969∼1.035
Disease duration	–0.022	0.008	7.635	0.006	0.978	0.963∼0.993
Primary school	–0.234	0.721	0.105	0.745	0.791	0.192∼3.251
Middle school	–0.003	0.728	0.000	0.996	0.997	0.239∼4.153
University degree or above	2.508	0.990	6.418	0.011	12.284	1.764∼85.490
History of stroke	–1.035	0.731	2.009	0.156	0.355	0.084∼1.488
Fazekas grade (2–3 grade)	0.705	0.591	1.421	0.233	2.023	0.635∼6.445
Constant	–0.037	1.589	0.001	0.982	0.964	0.043∼21.703

## Discussion

This study investigated the predictive value of baseline characteristics for cognitive function improvement after tDCS treatment by retrospectively analysing the clinical data of 123 PSCI patients who received tDCS therapy. Results of univariate analysis showed that there were significant differences in educational level, disease duration, Fazekas grading of white matter lesions, and history of stroke between the cognitive improvement group and the non-improvement group. Further multivariate logistic regression analysis confirmed that educational level and disease duration were independent predictive factors for significant improvement in cognitive function of PSCI patients after tDCS treatment.

### Educational Level: The Impact of Cognitive Reserve and Neuroplasticity on tDCS Efficacy

In this study, educational level emerged as an independent predictive factor for significant improvement in cognitive function after tDCS treatment, and this result is highly consistent with the “Cognitive Reserve Theory” [23]. This theory posits that individuals form more abundant neural connection networks and more efficient cognitive processing strategies through long-term education, and this reserve capacity can maintain or restore cognitive function through compensatory mechanisms after brain injury [24]. Its core lies in the richness and plasticity of neural circuits in the cerebral cortex—higher educational attainment is associated with denser synaptic connections in cognitive-related brain regions such as the prefrontal lobe and temporoparietal lobe, as well as higher redundancy of neuronal networks, providing more potential pathways for functional repair after brain injury [25]. Consistent with our findings, Li *et al*.’s study [26] indicated that higher cognitive reserve is associated with better cognitive outcomes. tDCS modulates cortical excitability through weak direct current, promotes the expression of neurotrophic factors, such as Brain-Derived Neurotrophic Factor (BDNF), and accelerates neural remodelling [27, 28]. Patients with higher educational levels may possess a stronger basis for neuroplasticity in the brain, making them more responsive to the excitability regulation induced by anodal stimulation. Through activating compensatory neural circuits and strengthening residual functional networks, they can achieve significant improvement in cognitive function.

From a clinical practice, these findings suggests that clinicians may prioritize recommending tDCS treatment for PSCI patients with higher educational levels, while it is crucial to avoid absolute judgments of “poor therapeutic effect” for those with lower educational levels. In the future, complementary cognitive reserve can be constructed for patients with lower educational levels through combined interventions such as cognitive training, thereby enhancing their sensitivity to tDCS treatment [23].

### Disease Duration: The Key Role of Early Intervention in Cognitive Function Recovery

Multivariate analysis revealed that for each additional day of disease duration, the probability of significant improvement in cognitive function after tDCS treatment in PSCI patients decreased by 2.2%, indicating that initiating tDCS treatment as early as possible after stroke onset is more likely to achieve significant cognitive improvement. Following stroke, the brain undergoes a dynamic process of “injury-repair-stabilization”: within 1–3 months after onset, a substantial number of “penumbra” neurons exist around the damaged brain area [29, 30]. These neurons are not completely necrotic but only in a state of functional inhibition. Meanwhile, the intracerebral inflammatory response gradually subsides and the blood-brain barrier permeability recovers, creating a favourable microenvironment for neural remodelling [31, 32]. Anodal stimulation of tDCS can directly enhance cortical excitability in the “penumbra” area, inhibit apoptotic signalling pathways, and promote angiogenesis and synaptic reconstruction, thereby rapidly improving cognitive function [33]. However, beyond approximately 3 months, glial scars gradually form in the brain injury area, neural circuits tend to stabilize, and neuroplasticity decreases significantly. As a result, tDCS can hardly effectively activate the repair mechanism, leading to weakened therapeutic effects. In addition, PSCI patients with longer disease duration may have formed a fixed pattern of cognitive impairment and are often accompanied by psychological problems such as anxiety and depression, which may further attenuate treatment response. Therefore, clinicians should attach importance to the early screening of PSCI, assess cognitive function as soon as possible after the stroke patient’s condition stabilizes, and promptly initiate tDCS treatment for those diagnosed with PSCI to maximize treatment benefits. For patients with prolonged disease duration, optimisation of treatment parameters (extending stimulation time and increasing treatment frequency) or combination with pharmacological and behavioural interventions may improve efficacy through multimodal interventions.

### Limitations

This study has the following limitations: (1) Retrospective design: Retrospective studies cannot avoid selection bias. All included patients were from a single centre, lacking validation from multi-centre and large-sample studies, which may lead to limitations in case selection and restrict the external validity of the results. Therefore, its external universality still needs further confirmation; (2) Sample size restriction: The relatively small sample size may limit the statistical power, stability and generalizability of the results, resulting in the failure to identify some potential predictive factors, especially insufficient statistical power for Fazekas grading of white matter lesions; (3) Lack of objective neuroimaging indicators: This study did not include neuroimaging parameters such as brain structure and brain function, making it impossible to explain the role of predictive factors more deeply from the perspective of neural mechanisms; (4) Some indicators in this study were evaluated using measurement tools, which may introduce information bias or measurement error.

## Conclusion

Through univariate and multivariate analyses, in this study, higher educational level and shorter disease duration may be independent predictive factors for significant improvement in cognitive function of PSCI patients after tDCS treatment. These findings provide important references for clinically screening suitable populations for tDCS therapy and facilitate the realization of individualized precision treatment for PSCI patients.

## Availability of Data and Materials

The data analysed is available upon request from the corresponding author.

## References

[1] He A, Wang Z, Wu X, Sun W, Yang K, Feng W (2023). Incidence of post-stroke cognitive impairment in patients with first-ever ischemic stroke: a multicenter cross-sectional study in China. The Lancet Regional Health. *Western Pacific*.

[2] Rost NS, Brodtmann A, Pase MP, van Veluw SJ, Biffi A, Duering M (2022). Post-Stroke Cognitive Impairment and Dementia. *Circulation Research*.

[3] Khan R, Devlin P, Urayama A, Ritzel RM (2025). Models and mechanisms of post-stroke dementia and cognitive impairment. *Frontiers in Stroke*.

[4] Kreiger K, Weiss E, Fluri F (2025). Novel therapies for post-stroke cognitive impairment: a systematic review. *Frontiers in Neurology*.

[5] Shen W, Fan X, Wang L, Zhang Y (2022). Traditional Chinese Medicine for Post-Stroke Cognitive Impairment: A Systematic Review and Meta-Analysis. *Frontiers in Pharmacology*.

[6] Zhang Y, Tang YW, Peng YT, Yan Z, Zhou J, Yue ZH (2024). Acupuncture, an effective treatment for post-stroke neurologic dysfunction. *Brain Research Bulletin*.

[7] Li Z, Yang L, Qiu H, Wang X, Zhang C, Zhang Y (2022). Comparative efficacy of 5 non-pharmacological therapies for adults with post-stroke cognitive impairment: A Bayesian network analysis based on 55 randomized controlled trials. *Frontiers in Neurology*.

[8] Fregni F, El-Hagrassy MM, Pacheco-Barrios K, Carvalho S, Leite J, Simis M (2021). Evidence-Based Guidelines and Secondary Meta-Analysis for the Use of Transcranial Direct Current Stimulation in Neurological and Psychiatric Disorders. *The International Journal of Neuropsychopharmacology*.

[9] Sloane KL, Hamilton RH (2024). Transcranial Direct Current Stimulation to Ameliorate Post-Stroke Cognitive Impairment. *Brain Sciences*.

[10] D’Amelio M, Di Lazzaro V (2023). Can transcranial magnetic stimulation rescue dopaminergic signalling in Alzheimer’s disease?. *Brain: a Journal of Neurology*.

[11] Liu L, Li Z, Zhou H, Duan W, Huo X, Xu W (2023). Chinese Stroke Association guidelines for clinical management of ischaemic cerebrovascular diseases: executive summary and 2023 update. *Stroke and Vascular Neurology*.

[12] Qu Y, Zhuo L, Li N, Hu Y, Chen W, Zhou Y (2015). Prevalence of post-stroke cognitive impairment in china: a community-based, cross-sectional study. *PloS One*.

[13] Cohen CI, Reisberg B, Yaffee R (2024). Global cognitive trajectory patterns in Alzheimer’s disease. *International Psychogeriatrics*.

[14] Song Y, Zeng L, Gao J, Chen L, Sun C, Yan M (2022). Adherence to High Dietary Diversity and Incident Cognitive Impairment for the Oldest-Old: A Community-Based, Nationwide Cohort Study. *Nutrients*.

[15] Santangelo R, Agosta F, Masi F, Spinelli EG, Cecchetti G, Caso F (2021). Plasma neurofilament light chain levels and cognitive testing as predictors of fast progression in Alzheimer’s disease. *European Journal of Neurology*.

[16] Joint Committee for Guideline Revision (2019). 2018 Chinese Guidelines for Prevention and Treatment of Hypertension-A report of the Revision Committee of Chinese Guidelines for Prevention and Treatment of Hypertension. *Journal of Geriatric Cardiology: JGC*.

[17] Guo L, Xiao X (2024). Guideline for the Management of Diabetes Mellitus in the Elderly in China (2024 Edition). *Aging Medicine (Milton (N.S.W))*.

[18] Srichawla BS, Barbini MK, Lessard D, Saczynski JS, McManus DD, Moonis M (2025). Fazekas score predicts cognitive decline & frailty in older adults: insights from the SAGE-AF cohort study. *Neurological Research and Practice*.

[19] Ohura T, Hase K, Nakajima Y, Nakayama T (2017). Validity and reliability of a performance evaluation tool based on the modified Barthel Index for stroke patients. *BMC Medical Research Methodology*.

[20] Woods AJ, Antal A, Bikson M, Boggio PS, Brunoni AR, Celnik P (2016). A technical guide to tDCS, and related non-invasive brain stimulation tools. *Clinical Neurophysiology: Official Journal of the International Federation of Clinical Neurophysiology*.

[21] Cheng XP, Wang ZD, Zhou YZ, Zhan LQ, Wu D, Xie LL (2024). Effect of tDCS combined with virtual reality for post-stroke cognitive impairment: a randomized controlled trial study protocol. *BMC Complementary Medicine and Therapies*.

[22] Creavin ST, Wisniewski S, Noel-Storr AH, Trevelyan CM, Hampton T, Rayment D (2016). Mini-Mental State Examination (MMSE) for the detection of dementia in clinically unevaluated people aged 65 and over in community and primary care populations. *The Cochrane Database of Systematic Reviews*.

[23] Passow S, Thurm F, Li SC (2017). Activating Developmental Reserve Capacity Via Cognitive Training or Non-invasive Brain Stimulation: Potentials for Promoting Fronto-Parietal and Hippocampal-Striatal Network Functions in Old Age. *Frontiers in Aging Neuroscience*.

[24] Sumowski JF, Rocca MA, Leavitt VM, Dackovic J, Mesaros S, Drulovic J (2014). Brain reserve and cognitive reserve protect against cognitive decline over 4.5 years in MS. *Neurology*.

[25] Stevens WD, Khan N, Anderson JAE, Grady CL, Bialystok E (2023). A neural mechanism of cognitive reserve: The case of bilingualism. *NeuroImage*.

[26] Li F, Kong X, Zhu H, Xu H, Wu B, Cao Y (2022). The moderating effect of cognitive reserve on cognitive function in patients with Acute Ischemic Stroke. *Frontiers in Aging Neuroscience*.

[27] Fridriksson J, Elm J, Stark BC, Basilakos A, Rorden C, Sen S (2018). BDNF genotype and tDCS interaction in aphasia treatment. *Brain Stimulation*.

[28] Chen Y, Mao L, Zhou Q, Bai D, Kong Y (2025). Role of BDNF-TrkB signaling in the improvement of motor function and neuroplasticity after ischemic stroke in rats by transcranial direct current stimulation. *Brain Research Bulletin*.

[29] Gauberti M, De Lizarrondo SM, Vivien D (2016). The “inflammatory penumbra” in ischemic stroke: From clinical data to experimental evidence. *European Stroke Journal*.

[30] Zeiler SR, Krakauer JW (2013). The interaction between training and plasticity in the poststroke brain. *Current Opinion in Neurology*.

[31] Levine DA, Galecki AT, Langa KM, Unverzagt FW, Kabeto MU, Giordani B (2015). Trajectory of Cognitive Decline After Incident Stroke. *JAMA*.

[32] Koukalova L, Chmelova M, Amlerova Z, Vargova L (2024). Out of the core: the impact of focal ischemia in regions beyond the penumbra. *Frontiers in Cellular Neuroscience*.

[33] Kubis N (2016). Non-Invasive Brain Stimulation to Enhance Post-Stroke Recovery. *Frontiers in Neural Circuits*.

